# Denosumab administration for bone metastases from solid tumors: a retrospective cross-sectional study

**DOI:** 10.1186/s12885-023-11495-w

**Published:** 2023-10-18

**Authors:** Kohei Mizuta, Hiromichi Oshiro, Ryo Katsuki, Yuichi Tsuha, Yusuke Aoki, Yasunori Tome, Kotaro Nishida

**Affiliations:** Department of Orthopedic Surgery, Graduate School of Medicine, University of the Ryukyus, 207 Uehara, Nishihara, Okinawa, 903-0215 Okinawa Japan

**Keywords:** Bone metastases, Denosumab, Skeletal-related events, Spinal instability neoplastic score, Mirels’ score

## Abstract

**Background:**

Little is known on how denosumab reduces skeletal-related events (SREs) by bone metastases from solid tumors. We sought to evaluate the effect of denosumab administration in patients with bone metastases from solid tumors.

**Methods:**

Data of patients treated with denosumab were collected from electronic medical charts (n = 496). Eligible participants in this study were adult patients (age ≥ 18 years) with metastatic bone lesions from solid tumors treated with denosumab. SREs, surgical interventions, the spinal instability neoplastic score (SINS) for spinal region, and Mirels’ score for the appendicular region were evaluated. To assess whether denosumab could prevent SREs and associated surgery, the SINS and Mirels’ score were compared between patients with and without SREs.

**Results:**

A total of 247 patients (median age, 65.5 years old; median follow-up period, 13 months) treated with denosumab for metastatic bone lesions from solid tumors were enrolled in this study. SREs occurred in 19 patients (7.7%). SREs occurred in 2 patients (0.8%) who took denosumab administration before SREs. Surgical interventions were undertaken in 14 patients (5.7%) (spinal and intradural lesions in five patients and appendicular lesions in nine patients). The mean SINS of patients without SREs compared to those with SREs were 7.5 points and 10.2 points, respectively. The mean Mirels’ scores of non-SREs patients and those with SREs were 8.07 points and 10.7 points, respectively. Patients with SREs had significantly higher Mirels’ score than non-SREs patients (p < 0.01). Patients with SREs had higher SINS than non-SREs patients (p = 0.09).

**Conclusions:**

SREs occurred in patients with higher SINS or Mirels’ scores. Two patients suffered from SREs though they took denosumab administration before SREs. Appropriate management of denosumab for patients with bone metastasis is significant. Surgical interventions may be needed for patients who with higher SINS or Mirel’s scores.

**Supplementary Information:**

The online version contains supplementary material available at 10.1186/s12885-023-11495-w.

## Background

Bone metastases often occur in advanced cancer patients, and cause skeletal-related events (SREs), including pathological fracture, paralysis from spinal cord compression, hypercalcemia, surgical intervention, and irradiation to bone. It results in a decline in performance status (PS), activities of daily living (ADL), and quality of life (QOL), and leads to difficulties in primary cancer treatment [1–3]. Moreover, pathological fractures due to SREs increase the risk of death [4]. Therefore, early therapeutic interventions for SREs are recommended and necessary.

Zoledronic acid and denosumab have been known as bone-modifying agents and are effective in delaying SREs in patients with bone metastases [5, 6]. These drugs are strongly recommended for bone metastases from breast cancers according to guidelines from the American Society of Clinical Oncology [7]. Denosumab is a fully human monoclonal antibody against the receptor activator of the nuclear factor-κB ligand, which suppresses osteoclast activation and prevents bone resorption and destruction [8]. It is currently available for bone metastases, osteoporosis, and giant cell tumors of the bone [9]. It has been reported that denosumab is more effective than zoledronic acid in preventing SREs in patients with bone metastases of solid tumors or multiple myeloma [10]. Although denosumab prevents or delays SREs in patients with bone metastases, [5, 10] no studies have reported how it affects surgical interventions in SREs.

Therefore, the purpose of this study was to assess the clinical outcomes of denosumab administrationon SREs for bone metastases originating from solid tumors.

## Patients and methods

### Study design and participants

This was a retrospective observational study conducted at a single institution. Eligible participants were adult patients (age ≥ 18 years), with metastatic bone tumors from solid tumors, and treated with denosumab between January 2012 and December 2020. We retrospectively extracted patient data for those treated with denosumab from electronic medical charts. Patients who switched from zoledronic acid to denosumab were excluded from this study. Those who were treated for giant cell tumors of the bone, bone metastases from primary bone and soft tissue sarcomas, and bone lesions from hematologic diseases were also excluded from this study. Moreover, those with incomplete data, < 3 months follow-up, as well as those who did not consent to participation were also excluded.

### Treatment procedure

Subcutaneous denosumab (120 mg) was administered every four weeks following a diagnosis of metastatic bone tumors. In some patients, physicians decided to extend the period of denosumab administration based on disease status. Denosumab was continued until unacceptable toxic effect, withdrawal of consent, or death.

### Procedures

SREs of this study were defined as pathological fractures, spinal cord compressions, surgery to bone, and hypercalcemia. Irradiation to bone was excluded from SREs in this study. Rates of SREs and surgical interventions were evaluated. In the spinal region, the spinal instability neoplastic score (SINS) [11] was evaluated. According to a previous study, a score of ≤ 6 was considered stable, 7–12 as moderate, and ≥ 13 as unstable [11]. In the appendicular region, Mirels’ score [12] was evaluated. Patients with scores greater than 8 points are considered for surgery [12]. The SINS and Mirels’ scores were assessed between SREs and non-SREs patients. Adverse events (AEs)-related to denosumab were also assessed. These included hypocalcemia, osteonecrosis of the jaw (ONJ), and atypical femoral fracture (AFF) [13]. In this study, hypocalcemia was defined based on a blood sample taken after the start of denosumab treatment that was below our standard albumin-adjusted calcium level, and ONJ was defined as an oral lesion involving exposure of the mandible or maxilla without prior head and neck radiation therapy. AFF was defined as per the revised diagnostic criteria from American Society for Bone and Mineral Research (2013) [14].

### Statistical analysis

Mann-Whitney U-test was used to compare the value of the SINS and Mirels’ score between SREs and non-SREs patients. Data are presented as mean ± standard deviation. Statistical analyses were performed with JMP version 15 (SAS institute inc., Cary, NC, U.S.A.). P < 0.05 was considered as significant differences.

## Results

### Patient characteristic

**Fig. 1:**
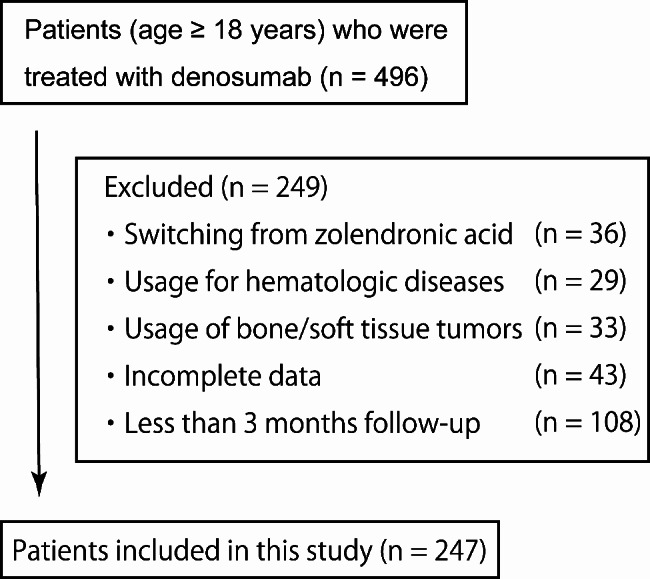
Flow diagram indicating study participation

A total of 496 patients treated with denosumab were screened from electronic medical charts. Of these, 249 patients were excluded due to switching zoledronic acid to denosumab (n = 36), usage for bone/soft tissue sarcoma (n = 33) and hematologic disease (n = 29), incomplete data collection (n = 43), and ≤ 3 months follow-up (n = 108). Finally, 247 patients treated with denosumab for bone metastases from solid tumors were included in this study (Fig. 1). Male patients were 157 (63.6%) and females were 90 (36.4%). The median age was 65.5 years (range 29–90). The median follow-up period was 13 months (range 3–85). Multiple bone metastases were observed in 170 patients (68.8%) and solitary bone metastasis was observed in 77 patients (31.2%); 124 patients (50.2%) received irradiation therapy for metastatic bone tumors. Primary solid tumors consisted of lung carcinoma in 71 patients (28.7%), breast and prostate carcinoma in 31 patients (12.5%), prospectively, renal cell carcinoma in 25 patients (10.1%), colorectal cancer in 21 patients (8.5%), and others in 68 patients (27.5%) (Table 1).

**Table 1:** Patient characteristics

Sex	
Men, n	157
Women, n	90
Age, median (range)	65.5 (29–90)
Follow-up (mos), median (range)	13.0 (3–85)
Bone metastases/metastasis	
Multiple, n	170
Solitary, n	77
Radiation therapy, n (%)	124 (50.2)
Primary	
Lung, n (%)	71 (28.7)
Breast, n (%)	31 (12.5)
Prostate, n (%)	31 (12.5)
Renal cell, n (%)	25 (10.1)
Colorectal, n (%)	21 (8.5)

n, number; mos, months

### SREs and denosumab treatment

SREs were observed in 19 patients (7.7%), in total (Table 2). Spinal SREs occurred in 5 patients (2.0%) and appendicular SREs occurred in 12 patients (4.9%) (femur in eight patients, humerus in three patients, and radius in one patient). Other region SREs occurred in 2 patients (0.4%) (clavicle and intradural). SREs included pains in seven patients, pathological fractures in seven patients, and spinal cord injuries in five patients. Denosumab administration was treated in two patients (0.8%) before SREs and seventeen patients (6.9%) after SREs. Surgical interventions were undertaken in 14 patients (5.7%) (Table 3). Spine /intradural surgeries were performed in five patients (2.0%). A posterior lumbar fusion was undertaken in four patients while a laminectomy was performed in one patient. Appendicular bone surgeries were performed in nine patients (3.6%). Wide excision and endoprosthetic arthroplasty as well as wide excision and intramedullary nail/plate fixation were respectively undertaken in two patients each. Arthroplasty was done in only four patients, and intramedullary nailing in one patient only.

**Table 2:** Skeletal-related events & rates

Total, n (%)	19 (7.7)
Location	
Appendicular, n (%)	12 (4.9)
Femur, n	8
Humerus, n	3
Radius, n	1
Spine, n (%)	5 (2.0)
Others, n (%)	2 (0.8)
Clavicle, n	1
Intradural, n	1
Type	
Pain, n (%)	7 (2.8)
Pathological fracture, n (%)	7 (2.8)
Paralysis, n (%)	5 (2.0)
Denosumab administration	
Before SREs, n (%)	2 (0.8)
After SREs, n (%)	17 (6.9)

n, number

**Table 3:** Surgical interventions for SREs

Total, n (%)	14 (5.7)
Location	
Spinal or intradural, n (%)	5 (2.0)
Posterior lumber fusion, n	4
Laminectomy, n	1
Appendicular, n (%)	9 (3.6)
Wide ex. & endoprosthetic arthroplasty, n	2
Wide ex. & IM nail/plate fixation, n	2
Arthroplasty, n	4
IM nail/plate fixation, n	1

ex, excision; IM, intramedullary; n, number; SREs, skeletal-related events

### SINS and Mirels’ score comparison between SRE and non-SRE patients

**Fig. 2:**
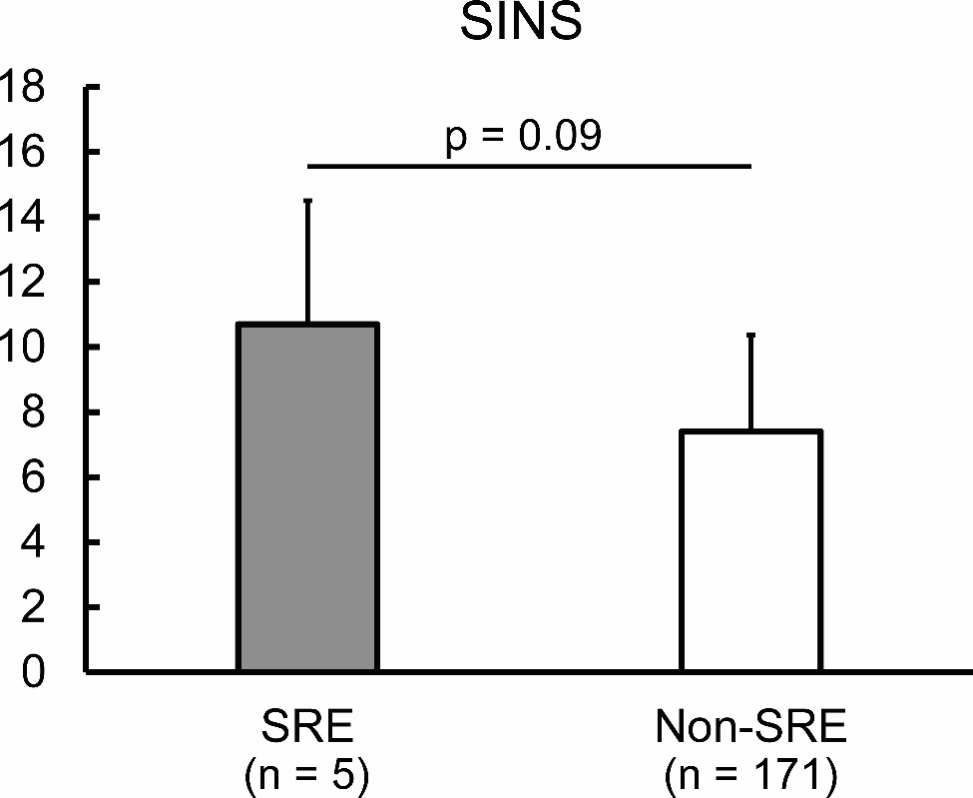
Comparison of spinal instabilities neoplastic scores (SINS) between skeletal-rerated event (SRE) and non-SRE group. Data are shown as mean ± standard deviation

**Fig. 3:**
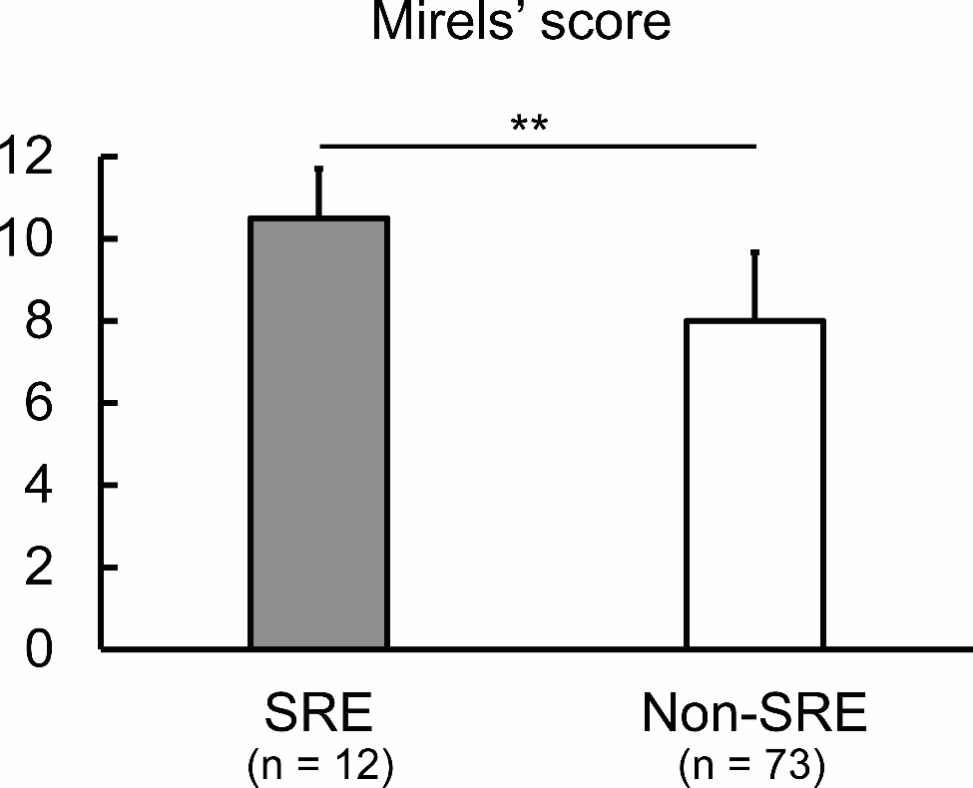
Comparison of Mirels’ score between skeletal-rerated event (SRE) and non-SRE group. Data are shown as mean ± standard deviation. **, p < 0.01 (Mann-Whitney U-test)

The mean SINS of non-SREs patients was 7.5 ± 2.9 (range, 2–15), while in SREs patients, it was 10.2 ± 3.7 (range, 2–15) (p = 0.09) (Fig. 2). The mean Mirels’ score was 8.1 ± 1.7 (range 4–12) in non-SREs patients and 10.7 ± 1.2 (range 9–12) in SREs patients (p < 0.01) (Fig. 3).

### Adverse events

Hypocalcemia occurred in 17 patients (6.9%). No patients developed ONJ in this study. One patient (0.4%) developed AFF probably due to prolonged administration of denosumab. This was treated with bilateral intramedullary nails. Denosumab was discontinued in 2 patients (0.8%) due to prolonged hypocalcemia.

## Discussion

In this study, we found that SREs occurrence rates under denosumab administration for metastatic bone lesions from solid tumors were 0.8%. In comparison, several studies have reported SREs occurrence rates of 11.5–14.4% [8, 15, 16] for similar indications. Therefore, our results in this regard were lower rate in comparison to those from previous reports.

Assessment of spinal instability is very important in determining the timing of surgery. The SINS is widely used to assess spinal instability in metastatic spinal tumors. A score of six or less is stable, 7–12 is moderate, and 13 or more is considered unstable [11]. The SINS has been shown to be reliable in many studies, but it is difficult to determine whether surgical intervention is necessary for moderate scores (range 7–12) [17–19]. It has been reported that more than 50% of patients with ≥ 10 points of SINS underwent stabilization and only 11% of patients with ≤ 9 points underwent instrumented fusion [20]. Moreover, Vargas et al. reported that patients with SINS of greater than 10 had increased surgery rates with a 1-year follow-up [21]. On the other hand, regarding radiation therapy, patients with higher SINS (median 10 points of SINS), and with spinal metastases, had increased rates of radiation failure [22]. Some reports revealed that prophylactic surgeries for spinal metastatic lesions could improve QOL, ADL, and PS [23, 24]. In this study, spinal/intradural SREs occurred in 6 patients (2.4%), and five patients (2.0%) underwent spinal surgery. The mean SINS score in SREs cases was 10.2 ± 3.7 (range 6–15). Given the above-mentioned risk of spinal surgery with higher SINS, prophylactic spinal surgeries may be needed for patients with higher SINS even though denosumab was administered.

On the other hand, Mirels’ score is used to assess for risk of pathological fracture of the extremities. Patients with scores greater than 8 points are considered for surgery [12]. A retrospective study reported that the mean Mirels’ score of patients who underwent prophylactic stabilization was 10.3 [25]. In this study, appendicular SREs occurred in 12 patients (4.9%), and nine patients (3.6%) underwent appendicular surgery. The mean Mirels’ score in the operated cases was 10.7 ± 1.2 (range 9–12). Given the similarities between our results and other reports, prophylactic surgeries may be needed for patients with higher Mirels’ scores as well as higher SINS even though denosumab was administered.

Hypocalcemia is a common electrolyte abnormality associated with denosumab. When compared with zoledronic acid, hypocalcemia occurred more frequently in denosumab (5.5–13%) than in zoledronic acid (3.4–6.0%) [8, 16, 26]. In this study, hypocalcemia occurred in 17 patients (6.9%). Denosumab has also been associated with occurrence of ONJ. In comparison with zoledronic acid, ONJ occurred 1.1–2.0% of patients administered denosumab and 1.0–1.4% of patients administered zoledronic acid [8, 16, 26]. In this study, no patients developed ONJ. Although the suppression of bone remodeling by bisphosphonate and denosumab is caused AFF, the pathogenesis of AFF is not well understood. A retrospective study reported that AFF occurred in 1.8% of patients with bone metastasis receiving denosumab [27]. In this study, AFF occurred in one patient (0.4%) in bilateral femurs, and bilateral intramedullary nailing was performed. Compared with previous reports, our results showed that denosumab administration for metastatic bone lesions from solid tumors was well-tolerated and AEs were manageable.

This study had several limitations. First, it was a retrospective study at a single facility. Second, it consisted of various types of cancers. Therefore, the efficacy of denosumab, which may differ depending on the type of cancer, could not be evaluated accurately for specific types of cancer. Additionally, the interval/duration of denosumab administration was decided based on the treating physician’s discretion. Third, some patients underwent radiotherapy while others did not. This may have confounded the real results of denosumab administration alone. Fourth, this study did not perform comparative analyses with patients who did not receive denosumab. It is widely known that denosumab can reduce the incidence of SREs and improve the overall survival. This study demonstrated, for the first time, that patients with high SINS or Mirels’ score may be at risk of SREs despite being administered denosumab. Fifth, this study focused on the risk of SREs in patients under denosumab administration and had a short-term follow-up period. Further investigation on its benefits regarding the quality of life, with a longer follow-up period, may be warranted. In addition, multi-center prospective studies would be needed to determine whether prophylactic surgeries could improve clinical outcomes for patients with high SINS or Mirels’ score in the future. However, our study revealed that denosumab administration was beneficial for patients with SINS or Mirels’ score < 9 to prevent SREs and associated surgeries.

## Conclusions

Denosumab was beneficial in the treatment of metastatic bone lesions from solid tumors. It is important for patients with bone metastasis to take appropriate denosumab administration before SREs. Patients with higher SINS or Mirels’ scores may need prophylactic surgeries to prevent from SREs.

### Electronic supplementary material

Below is the link to the electronic supplementary material.

Supplementary Material 1

## Data Availability

All data generated or analyzed during this study are included in this published article and its supplementary information files.

## Abbreviations

ADL: Activities of daily living
AEs: Adverse events
AFF: Atypical femoral fracture
ONJ: Osteonecrosis of the jaw
PS: Performance status
QOL: Quality of life
SINS: Spinal instability neoplastic score
SREs: Skeletal-related events
